# Interaction and Modulation of Two Antagonistic Cell Wall Enzymes of Mycobacteria

**DOI:** 10.1371/journal.ppat.1001020

**Published:** 2010-07-29

**Authors:** Erik C. Hett, Michael C. Chao, Eric J. Rubin

**Affiliations:** 1 Department of Molecular Biology, Massachusetts General Hospital, Boston, Massachusetts, United States of America; 2 Department of Microbiology and Molecular Genetics, Harvard Medical School, Boston, Massachusetts, United States of America; 3 Department of Immunology and Infectious Diseases, Harvard School of Public Health, Boston, Massachusetts, United States of America; Johns Hopkins School of Medicine, United States of America

## Abstract

Bacterial cell growth and division require coordinated cell wall hydrolysis and synthesis, allowing for the removal and expansion of cell wall material. Without proper coordination, unchecked hydrolysis can result in cell lysis. How these opposing activities are simultaneously regulated is poorly understood. In *Mycobacterium tuberculosis*, the resuscitation-promoting factor B (RpfB), a lytic transglycosylase, interacts and synergizes with Rpf-interacting protein A (RipA), an endopeptidase, to hydrolyze peptidoglycan. However, it remains unclear what governs this synergy and how it is coordinated with cell wall synthesis. Here we identify the bifunctional peptidoglycan-synthesizing enzyme, penicillin binding protein 1 (PBP1), as a RipA-interacting protein. PBP1, like RipA, localizes both at the poles and septa of dividing cells. Depletion of the *ponA1* gene, encoding PBP1 in *M. smegmatis*, results in a severe growth defect and abnormally shaped cells, indicating that PBP1 is necessary for viability and cell wall stability. Finally, PBP1 inhibits the synergistic hydrolysis of peptidoglycan by the RipA-RpfB complex *in vitro*. These data reveal a post-translational mechanism for regulating cell wall hydrolysis and synthesis through protein–protein interactions between enzymes with antagonistic functions.

## Author Summary

Bacteria have a complex problem to solve. On one hand, they need to hydrolyze existing and synthesize new cell wall to allow for cell expansion during growth. On the other hand, they need to maintain a continuous layer of cell wall to preserve shape and protect from osmotic lysis. To do this, bacteria must tightly coordinate the processes of cell wall hydrolysis and synthesis. How these opposing activities are simultaneously regulated is poorly understood. We previously demonstrated the interaction between two cell wall hydrolytic proteins, RpfB and RipA, in mycobacteria. This RpfB-RipA complex resulted in enhanced hydrolysis of cell wall, suggesting protein–protein interactions as one mechanism for regulating hydrolysis. However, what regulates interactions of these potentially lethal enzymes and what coordinates hydrolysis with synthesis remains unknown. To investigate this question, we screened for mycobacterial proteins that interact with, and thus potentially regulate, RipA. Here, we report the interaction of RipA with PBP1, a cell-wall-synthesizing enzyme. Depletion of PBP1 from mycobacteria results in misshapen cells and impaired growth. Moreover, we find that PBP1 inhibits the synergistic activity of the RipA-RpfB interaction. These data reveal a mechanism for coordinating cell wall hydrolysis and synthesis through interactions between antagonistic enzymes.

## Introduction

*Mycobacterium tuberculosis*, the causative agent of tuberculosis, kills approximately two million people each year and remains dormant within an estimated one-third of the world's population [1]. *M. tuberculosis* has the remarkable ability to survive extended periods of time under stressful conditions within the host, only to reactivate, grow, and cause a relapse into active disease [2]. Reactivation likely relies upon the ability of mycobacteria to regulate the expansion and remodeling of cell wall material, an essential yet poorly understood bacterial process. Because cell wall biology is a rich area for antibiotic development, elucidating the mechanisms of essential cell wall processes in mycobacteria offers new avenues for chemotherapy targeted to actively growing or reactivating bacteria. Mycobacteria possess basic cell wall remodeling requirements similar to other bacteria, such that understanding mycobacterial cell wall homeostasis may provide new insights into universal paradigms of cell wall regulation.

One such highly conserved area of cell wall remodeling is the need for regulation of peptidoglycan synthesis and degradation. Peptidoglycan (PG) is found in nearly all bacteria and is responsible for giving bacteria their shape and structural integrity [3], [4]. *Escherichia coli* PG is composed of polysaccharides containing repeating disaccharide subunits of N-acetyl glucosamine and N-acetyl muramic acid, while mycobacterial PG contains N-acetyl glucosamine and a mixture of N-glycolyl muramic acid and N-acetyl muramic acid [5]. These polymers are cross-linked by peptide bridges into a rigid three-dimensional lattice known as a sacculus. PG elongation requires a suite of enzymes with both synthetic and hydrolytic activities. Bifunctional penicillin binding proteins (PBPs) possess both transglycosylase and transpeptidase domains that covalently incorporate newly synthesized PG polymers into the existing sacculus. To accomplish this in *E. coli*, hydrolytic enzymes with lytic transglycosylase and endopeptidase specificity are thought to first remove old PG monomers from the cell wall before incorporation of a new three-unit PG polymer [6]. Little is known about how mycobacteria expand and degrade their septal and polar PG. While it is useful to consider how other bacteria metabolize PG, it has yet to be shown if these models hold true for mycobacteria.

The coordination of PG synthases and hydrolases (also known as autolysins) is critical for growth and division, as well as maintenance of cellular structural integrity. Thus, a mechanism for controlling cell wall hydrolases must exist, yet the molecular details of this process are not well defined. Protein-protein interactions are potentially a central element of autolysin homeostasis, since binding partners can inhibit, sequester, or activate other proteins. Multiple interactions have been found between PG synthetic and hydrolytic enzymes in *Haemophilus influenzae* and *E. coli*, leading to the hypothesis that these remodeling enzymes may exist as holoenzyme complexes *in vivo* [7], [8], [9]. Despite these biochemical characterizations, the functional consequence of these interactions remains largely unknown.

In mycobacteria, as in other bacteria, the regulation of PG remodeling is poorly understood. Recent work in mycobacteria proposes a PBP3-FtsW-FtsZ complex that regulates the initiation of septation, but little is known how PG synthesis and hydrolysis are coordinated during this event [10]. We have previously shown that the *Mycobacterium tuberculosis* endopeptidase RipA (Rv1477) interacts with a lytic transglycosylase, RpfB, at the septum of dividing cells [11]. This interaction positively regulates PG hydrolysis since the RipA-RpfB complex synergistically degrades PG *in vitro* [12]. Since RipA is essential for division in *M. smegmatis* and *M. tuberculosis* [12], [13], this endopeptidase is an attractive target for studying regulation of cell wall homeostasis. While the interaction of RipA and RpfB appears to be functionally important, it is unclear how two enzymes that both degrade PG could, by themselves, regulate PG metabolism. To this end, we investigated how additional protein-protein interactions may modulate RipA function.

Here, we identify the mycobacterial bifunctional PG synthesizing enzyme, penicillin-binding protein 1 (PBP1), as a regulator of RipA-RpfB PG hydrolase activity. We report that PBP1 interacts with RipA in a yeast two-hybrid assay and co-precipitates with RipA. PBP1 localizes to the poles and septa, the sites of PG synthesis in mycobacteria and depletion of the PBP1 gene, *ponA1*, from mycobacteria results in the cessation of growth and formation of abnormally shaped, structurally compromised cells. Finally, PBP1 inhibits the synergistic hydrolysis of PG by the RipA-RpfB complex. Together, these data support a model where PBP1 restrains RipA-RpfB cell wall degradation in mycobacteria through a novel protein-protein interaction between antagonistic proteins.

## Results

### Yeast two-hybrid screen using *M. tuberculosis* RipA identifies PBP1

RipA was previously identified through a screen for mycobacterial proteins that interact with RpfB, and was shown to be a PG hydrolase necessary for cell division [11], [12]. We hypothesized that additional factors may interact with and regulate the activity of RipA during coordinated cell division and growth. Therefore, we conducted a yeast two-hybrid screen to identify novel RipA interacting proteins.

**Figure 1:**
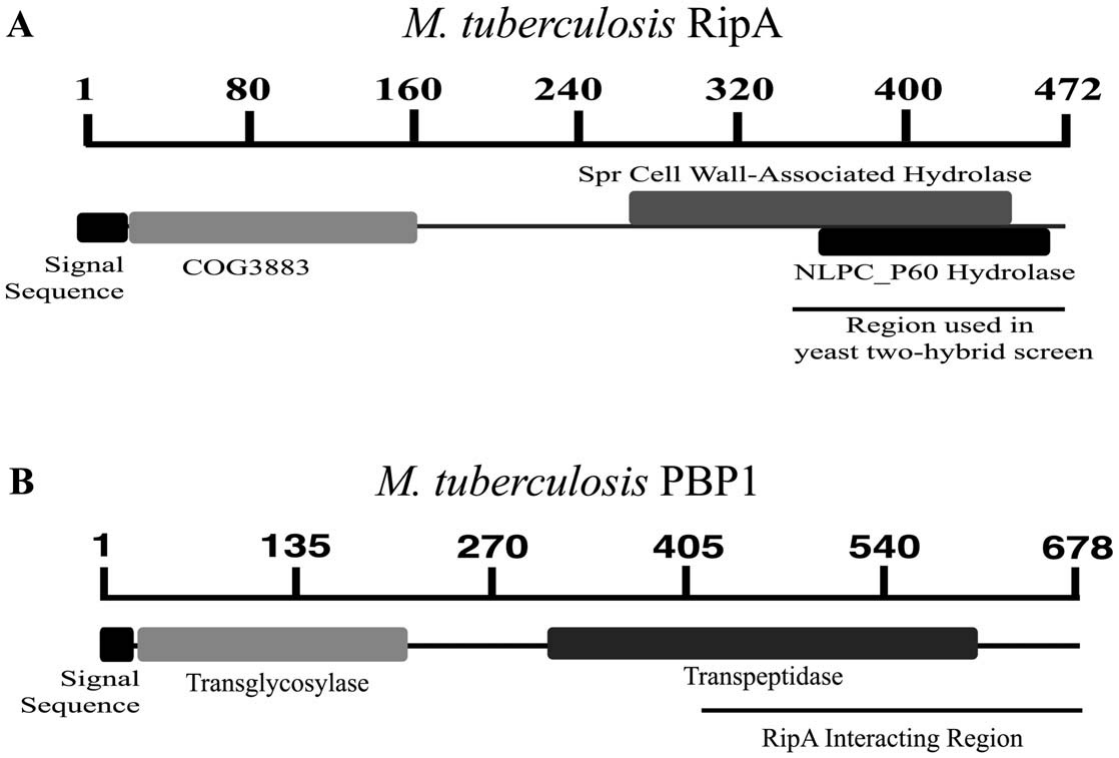
*M. tuberculosis* RipA identifies bifunctional synthase PBP1. (A) RipA, a 472 residue protein, contains a domain of unknown function (COG3883) as well as a predicted endopeptidase domain. The region used to screen for interacting proteins in a yeast two-hybrid screen is shown, consisting of amino acids 350–472. (B) PBP1, a 678 residue protein encoded by *ponA1*, is a bifunctional peptidoglycan synthase. PBP1 contains a transglycosylase domain at the N-terminus and a penicillin-sensitive transpeptidase domain at the C-terminus.

A translational fusion was made between the C-terminal 123 amino acids of *M. tuberculosis* RipA (Figure 1A) and the GAL4 DNA binding domain (BD-RipA), and screened against a random library of *M. tuberculosis* genomic fragments translationally fused to the GAL4 activation domain (AD). Approximately 1×10^6^ independent clones were screened for interaction with RipA by histidine prototrophy and β-galactosidase activity. Potential interactors were counterscreened for non-specific interactions and evaluated by quantitative β-galactosidase assays. From this screen, we identified a region encoding the C-terminal 259 amino acids of penicillin-binding protein 1 (PBP1) that interacts with RipA (Figure 1B).

### Mycobacterial PBP1 and RipA interact at their C-terminal domains

Mycobacterial PBP1 is a high molecular weight, class A, penicillin-binding protein [14]. The N-terminus of PBP1 contains a noncleavable signal sequence by which PBP1 is translocated across and anchored to the plasma membrane [14]. The N-terminus also contains a transglycosylase domain homologous to the *E. coli* PBP1 [15] and is responsible for ligating N-glycolyl muramic acid from existing PG sacculus to N-acetyl glucosamine from lipid II PG precursor monomers (Figure 1B). The C-terminus of mycobacterial PBP1 contains a penicillin binding transpeptidase domain homologous to the *E. coli* PBP1 [15] that crosslinks D-alanine to the dibasic amino acid D-meso-diaminopimelic acid (DAP) between two parallel strands of PG.

**Figure 2:**
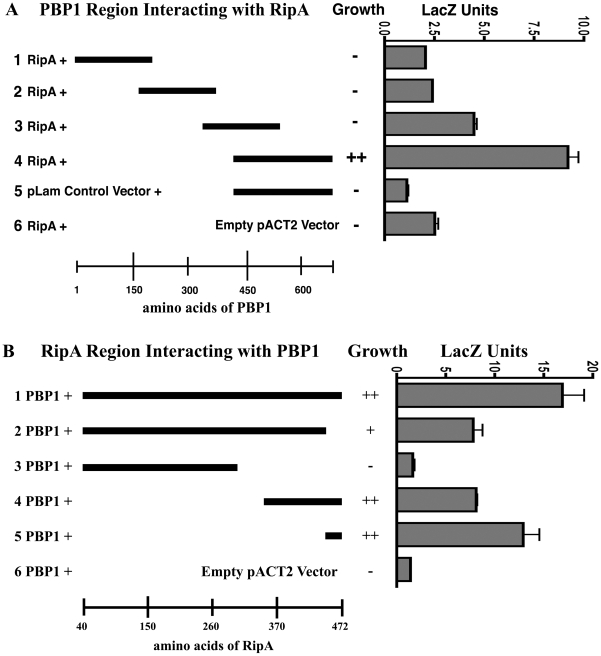
C-terminal regions are required for interaction. (A) Data from quantitative LacZ assays of four overlapping regions of 200 amino acids each of PBP1 tested for interaction with the C-terminal 123 amino acids of RipA. The C-terminal 259 amino acids of PBP1 were found to be sufficient for interaction with RipA, while regions lacking the C-terminal 150 amino acids failed to interact. Data shown are from a representative experiment done in triplicate. Data are represented as mean +/− SEM. (B) Data from quantitative LacZ assays of several different deletion constructs for interaction with the C-terminal 259 amino acids of PBP1 in the yeast two-hybrid assay. Deletion of the C-terminal of RipA decreased the intensity of interaction and the C-terminal 25 amino acids of RipA were sufficient for interaction with PBP1. Data shown are from a representative experiment done in triplicate. Data are represented as mean +/− SEM.

To determine the interaction domains of mycobacterial PBP1 and RipA, we mapped the interacting regions of each protein using the yeast two-hybrid system. Four overlapping regions of 200 amino acids each of PBP1 were assayed for interaction with the C-terminal 123 amino acids of RipA. Of the constructs tested, only the construct containing the C-terminal 259 amino acids of PBP1 was sufficient for interaction with RipA, while constructs lacking the C-terminal 150 amino acids failed to interact (Figure 2A). This interacting region contains two-thirds of the transpeptidase domain of PBP1.

In concert, we created and tested several RipA deletion constructs for interaction with the C-terminal 259 amino acids of PBP1 in the yeast two-hybrid assay. Deletion of the extreme C-terminus of RipA abrogated the interaction (Figure 2B, lanes 2&3), while the C-terminal 25 amino acids of RipA were sufficient for binding PBP1 (Figure 2B, lane 5). This region is adjacent to the predicted endopeptidase domain of RipA and, interestingly, has been shown to bind RpfB [11]. These results demonstrate that RipA and PBP1 interact at the domains that are responsible for cleaving and forming, respectively, the crosslinks between PG strands. Since the RipA interaction domain also binds the PG hydrolase domain of RpfB, PBP1 and RpfB may participate in concert to regulate septal PG remodeling.

### Mycobacterial PBP1 and RipA coprecipitate

**Figure 3:**
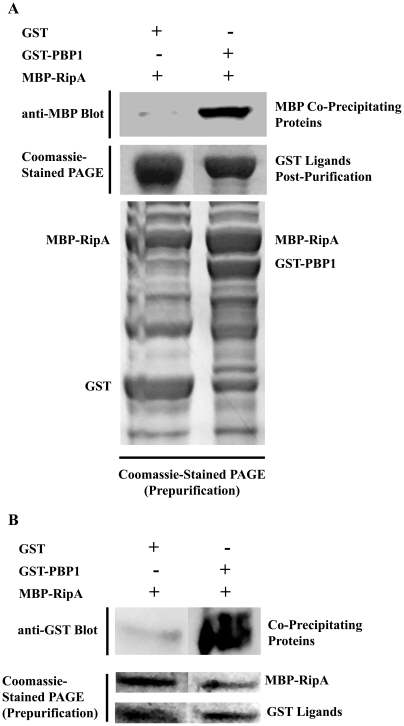
Recombinant PBP1 coprecipitates with RipA *in vitro*. (A) Fusion proteins were co-expressed in *E. coli* and GST fusion proteins were purified directly from the lysate. Co-purifying MBP fusion proteins were detected by Western blotting using anti-MBP antibody (top panel). Unfused GST was used to test the specificity of the interaction. A Coomassie-stained PAGE gel containing lysates obtained prior to GST purification (bottom panel) and after GST purification (middle panel) is shown to demonstrate that similar amounts of proteins were available for pulldown. (B) Proteins were separately purified from *E. coli*, combined as indicated in equimolar amounts, incubated, then purified on amylose resin. Samples were taken before (middle and bottom panels) and after (top panel) MBP purification. A Coomassie-stained PAGE gel containing protein mixtures prior to MBP purification is shown to demonstrate that similar amounts of proteins were available for pulldown. Co-purifying GST fusion proteins were detected by Western blotting using anti-GST antibody. Unfused GST was used to test the specificity of the interaction.

To confirm the specific interaction of RipA and PBP1, we performed an *in vitro* co-precipitation assay. Translational fusions of the C-terminal 259 amino acids of *M. tuberculosis* PBP1 with glutathione-S-transferase (GST), as well as the C-terminal 283 amino acids of *M. tuberculosis* RipA with maltose-binding protein (MBP) were constructed. *E. coli* co-expressing either GST and MBP-RipA or GST-PBP1 and MBP-RipA were lysed, and GST fusion proteins were purified using glutathione sepharose resin. Co-purifying proteins were detected by Western blotting with an anti-MBP antibody (top panel). Coomassie Blue-staining demonstrated that the amounts of GST (middle panel) and MBP available were similar (bottom panel). We observed that MBP-RipA is only detected when GST-PBP1 was present. These results demonstrate that RipA co-purifies specifically with PBP1, and has no detectable interaction with GST (Figure 3A) despite precipitating the same amount of recombinant GST. To control for the amount of protein available for interaction and precipitation, GST-PBP1, GST, and MBP-RipA were expressed separately and purified. GST and MBP-RipA or GST-PBP1 and MBP-RipA were mixed in equimolar amounts, incubated, and MBP tagged proteins precipitated with amylose resin. Co-purifying proteins were detected by Western blotting with an anti-GST antibody. Again we observed that similar amounts of GST (middle panel) and MBP (bottom panel) were purified using Coomassie Blue staining. We found that GST-PBP1 co-purified with MBP-RipA, but GST alone did not (Figure 3B). Taken together, this work demonstrates that RipA and PBP1 specifically interact *in vivo* in bacteria, *in vitro*, and in a yeast two-hybrid system.

### PBP1 localizes to the poles and septa of mycobacteria

**Figure 4:**
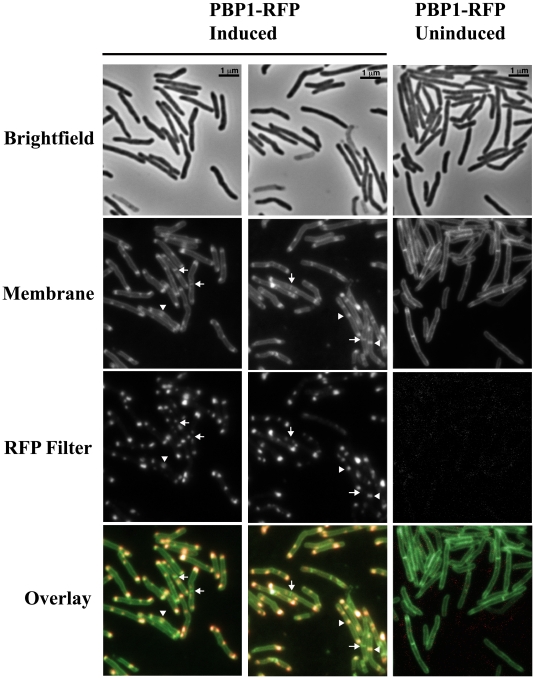
PBP1 localizes to the poles and septa of *M. smegmatis*. Fluorescence microscopy of *M. smegmatis* expressing *M. tuberculosis* PBP1 fused to monomeric red fluorescent protein (RFP). PBP1-RFP fusion protein is under the control of a tetracycline-inducible promoter. PBP1 localized to the poles and also to septa. Arrows indicate narrow septa where PBP1 does not localize and arrowheads indicate larger septa where PBP1 localizes.

In most other bacteria, PG synthesizing proteins, including PBPs, localize to the septum and/or lateral cell walls, indicating a role in septal and/or lateral wall PG synthesis, respectively [16], [17], [18]. To determine where PBP1 regulates PG remodeling, we assessed its localization *in vivo*. *M. tuberculosis* PBP1 was fused at its C-terminus to monomeric red fluorescent protein (RFP) and expressed under the control of a tetracycline-inducible promoter. We found that *M. tuberculosis* PBP1-RFP predominantly localized to the poles of *M. smegmatis* (the site of cell growth in mycobacteria) and occasionally at the septa of dividing cells (Figure 4). PBP1-RFP was not toxic, as cells expressing the protein exhibited normal morphology and growth (data not shown). Uninduced PBP1-RFP yielded no detectable fluorescence and RFP alone remained diffuse and cytosolic, with no observable bands of localization (data not shown), demonstrating that the PBP1-RFP signal is specific. Thus, PBP1 localizes to the poles and septa of mycobacteria, in contrast to the septal-only localization described for *B. subtilis* [18], [19]. This finding is consistent with evidence indicating that mycobacteria grow from their tips [20], [21].

### Depletion of mycobacterial PBP1 blocks normal cell division

**Figure 5:**
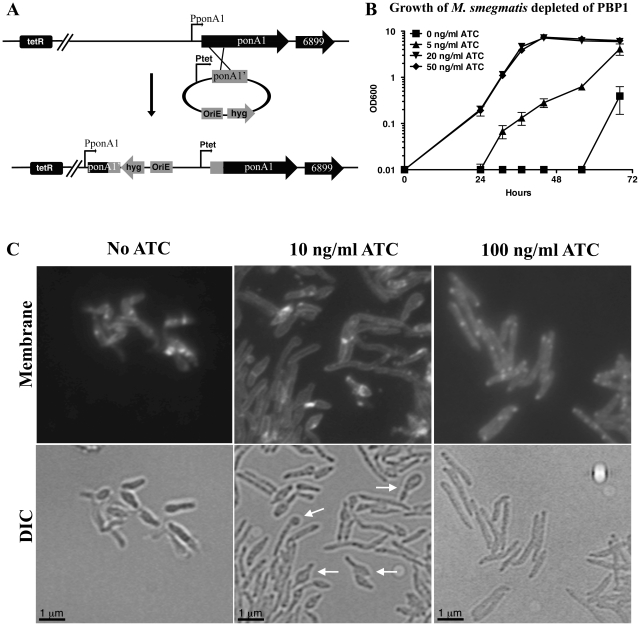
Depletion of PBP1 blocks cell division. (A) Diagram showing the strategy used to replace the native promoter of the *ponA1* operon (PponA1) in *M. smegmatis* with a tetracycline-inducible promoter (Ptet) through homologous recombination (strategy and diagram adapted from [12]). OriE: *E. coli* origin of replication. (B) PBP1 (*ponA1*) depletion strain of *M. smegmatis* was grown with inducer (ATC = anhydrotetracycline), then inoculated into media with decreasing amounts of inducer and followed by OD_600_ over time. Data are represented as mean +/− standard deviation. (C) Micrographs of *M. smegmatis* PBP1 (*ponA1*) depletion strain with membranes imaged by staining with TMA-DPH. Bacteria were grown with no anhydrotetracycline inducer (No ATC), 10, or 100 ng/ml ATC. Bacteria grown with 100 ng/ml ATC grew as wildtype, while No ATC and 10 ng/ml ATC grew slowly, with bulbous poles and round-shaped regions (indicated by white arrows). Bacteria were visualized with a 100× objective.

Given its localization *in vivo*, it is likely that PBP1 is involved in both elongation and septation. Strains of *E. coli* with both *ponA1* homologues deleted are nonviable [22] and null strains of *ponA1* homologues in the actinobacterium *C. glutamicum* are defective for elongation and septation [23]. To determine the functional role of PBP1 in mycobacteria, we constructed a conditional depletion strain in *M. smegmatis*, where transcription of the genes encoding PBP1 *(MSMEG6900)* and its operon (Figure S1) are activated by an inducible tetracycline promoter (Figure 5A). Since we hypothesized that PBP1 is involved in PG synthesis and cell division, we expected that depletion of PBP1 should yield changes in morphology and/or viability.

When the conditional PBP1 depletion strain was grown in the absence of inducer, we observed a dramatic growth defect (Figure 5B). The PBP1 conditional strain grew normally in the presence of inducer and was impaired for growth in the absence of inducer in a dose-dependent manner. Due to the high selective pressure against depletion of PBP1, cultures without inducer appeared to recover and grow at late time points (Figure 5B). However, this was due to escape from repression, as cells from these late time points were no longer depleted for *ponA1* transcription (Figure S2). The observed growth defect correlated with gross morphological changes. PBP1-operon depletion led to single, short, unseptated cells, suggesting that PBP1 functions in both elongation and septation. Furthermore, these cells possessed bulbous regions, commonly at their ends (Figure 5C, arrows), which is indicative of increased cell wall hydrolysis and loss of structural and morphological integrity. This is consistent with a model of cell wall regulation by PG synthase-hydrolase complexes.

**Figure 6:**
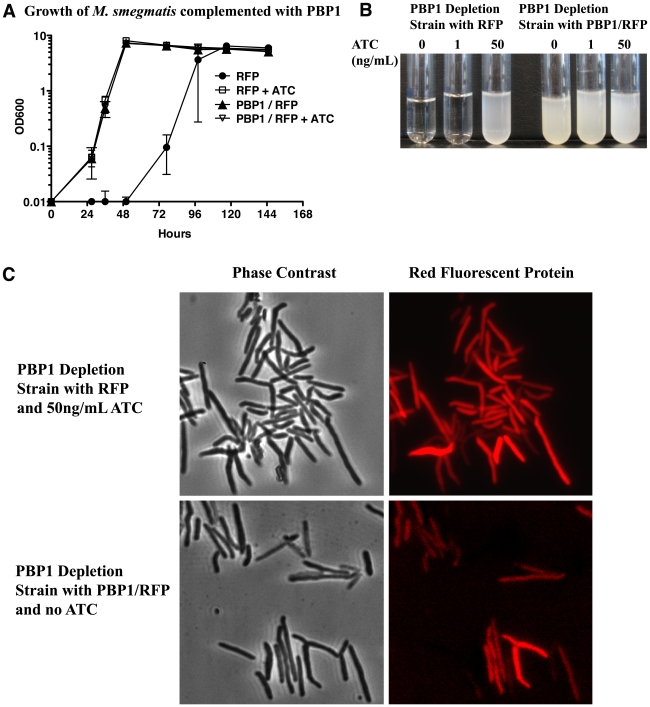
*M. smegmatis ponA1* complements *ponA1* operon depletion. (A) The *M. smegmatis ponA1* depletion strain was complemented with either a vector expressing an operon of *M. smegmatis ponA1* and red fluorescent protein (RFP) or RFP alone. Complemented strains were grown in the presence (50 ng/ml ATC) or absence of inducer and growth was determined by OD_600_. While the RFP complemented strain only grew in the presence of inducer, the PBP1 complemented strain grew normally in both the absence and presence of inducer. (B) At 36 hours post-induction, cultures from RFP and *ponA1* complemented strains were photographed. Data is representative of several biological replicates for both strains. (C) Microscopic analysis of complemented strains—either the RFP complemented strain grown with inducer or the *ponA1* complemented strain without inducer—showed that both strains expressed RFP from the complementation vector and appear morphologically normal.

To confirm that the growth defect and morphological abnormalities observed under conditions lacking inducer were specifically due to PBP1 depletion (as opposed to polar effects on the downstream genes in the operon), we complemented the PBP1 conditional strain with either a vector containing a constitutive promoter expressing *M. smegmatis* PBP1 or an empty vector. Both constructs contain the gene encoding red fluorescent protein (RFP) as a control for expression from the complementation plasmid. As shown in Figure 6A & B, the PBP1 depletion strain does not grow in the absence of inducer unless a plasmid producing PBP1 is provided *in trans*. This complementation also applies to the observed morphological defect. When the conditional mutant is complemented with PBP1 and grown in the absence of inducer, the cells are morphologically identical to cells of the conditional mutant without the complementing plasmid grown in the presence of inducer (Figure 6C). Both strains produced RFP, demonstrating that PBP1 is expressed off the complementation vector.

### PBP1 inhibits the synergistic hydrolysis of cell wall by RipA and RpfB

RipA can hydrolyze peptidoglycan as shown in studies using a variety of FITC-labeled, cell wall-derived substrates [12]. RipA hydrolytic activity is augmented in the presence of RpfB. Given that RipA binds PBP1 with the same domain sufficient for RpfB interaction, we sought to determine whether PBP1 affects the PG hydrolytic activity of RipA alone or in complex with RpfB.

**Figure 7:**
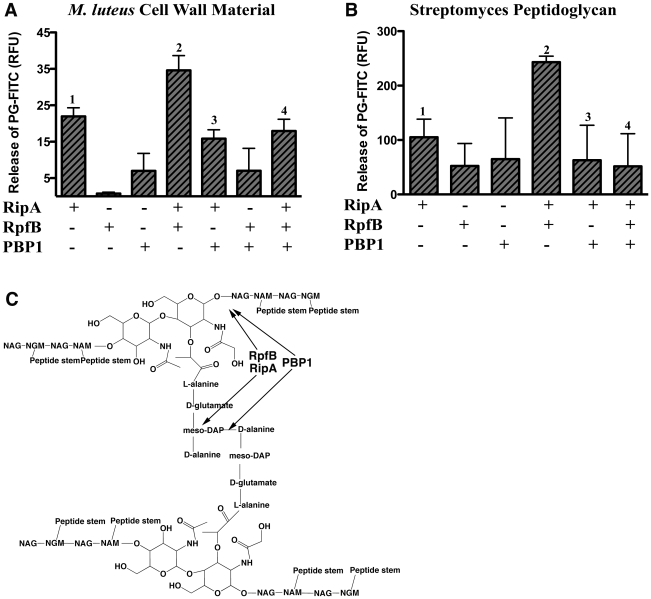
PBP1 inhibits the synergistic hydrolysis of cell wall by RipA-RpfB complex. N-terminal GST fusion proteins were expressed and purified from *E. coli*. Equal amounts of individual or combinations of proteins were incubated with insoluble FITC-labeled substrate: *M. luteus* cell wall material (A) or *Streptomyces* peptidoglycan (B). The extent of hydrolysis was determined by measuring the amount of soluble FITC remaining after centrifugation, and thus released during hydrolysis of the insoluble substrate. GST and buffer alone were used to determine background release of FITC and were subtracted from final values. Data are from representative experiments, each done in triplicate. Data are represented as mean +/− SEM. p-values for one-tailed, unpaired t-tests: (A) 1 vs. 2: 0.027 significant, 2 vs. 4: 0.016 significant, 1 vs. 3: 0.074 not significant, 1 vs. 4: 0.188 not significant, (B): 1 vs. 2: 0.009 significant, 2 vs. 4: 0.018 significant, 1 vs. 3: 0.279 not significant, 1 vs. 4: 0.240 not significant (significant to p<0.05). (C) Schematic diagram of the basic structure of mycobacterial peptidoglycan, indicating where RpfB and RipA are predicted to have hydrolytic activity and where PBP1 is predicted to have synthetic activity. Lines connecting NAG to NGM represent β-1,4-glycosidic bonds, while lines connecting NGM to NGM represent peptide cross-linkages. NAG: N-acetyl glucosamine, NGM: N-glycolyl muramic acid, NAM: N-acetyl muramic acid (note that mycobacteria have a mixture of NGM and NAM, with the NGM structure shown here).

We expressed and purified PBP1, RipA, and RpfB as GST fusion proteins in *E. coli*, and incubated various combinations of these enzymes with several FITC-labeled cell wall-derived substrates. Confirming our previous results, RipA, but not GST alone, was able to hydrolyze peptidoglycan purified from *Streptomyces*, a substrate that structurally resembles PG derived from mycobacteria. Furthermore, synergistic hydrolysis was observed when RpfB was combined with RipA, as previously shown [12]. However, addition of PBP1 to a reaction containing both RipA and RpfB inhibited this synergy, resulting in activity levels at or below that of RipA alone (Figure 7A, B). The activity of RipA combined with PBP1 was similar to that of RipA alone, suggesting that the PBP1 interaction does not affect the endogenous activity of RipA, but rather modulates the hydrolytic potential of the RipA-RpfB complex. As expected, PBP1 alone did not show appreciable activity above background, nor did addition of PBP1 to RpfB. These results demonstrate that PBP1 is able to modulate cell wall hydrolytic activity by inhibiting the synergistic interaction between RpfB and RipA.

## Discussion

How new peptidoglycan is coordinately synthesized and hydrolyzed during bacterial growth and division is not well understood. Both growth and division are dependent upon PG hydrolases and synthases working in a spatially and temporally coordinated manner. During cell division in mycobacteria, a thick layer of septal PG is initially synthesized between the two daughter cells before being digested by hydrolases. This results in two new poles on separate daughter cells [24], [25]. Likewise during cell elongation, existing PG is thought to be hydrolyzed to allow new PG subunits to be incorporated. While little is known about how mycobacteria regulate these events, some general concepts can be gleaned from other bacteria. In *E. coli* and *H. influenzae*, evidence suggests that there are PG-synthesizing and degrading complexes assembled for PG elongation and midcell septation in bacteria [7], [8], [26]. For instance, PBP1B interacts with MltA, a lytic transglycosylase, and MipA, a structural protein [8], comprising part of a theorized larger complex. These data and other studies identifying complexes containing PG-synthesis enzymes [7], [8], [26] strengthen the concept that PG-remodeling holoenzymes exist and may consist of as much as four enzymatic domains including opposing transpeptidase and endopeptidase, and transglycosylase and lytic transglycosylase, activities. A complex of these four functions should be sufficient to insert and remove PG monomers during elongation or septation in *E. coli* [27], [28]; however, no report to date has identified this theoretical complex in any bacteria. Here we demonstrate the interaction of three mycobacterial proteins – RpfB, RipA and PBP1 – containing a combination of domains that fulfills the four theoretically necessary reactions for PG remodeling.

Recently, functional data has emerged to show that protein-protein interactions between PG modifying enzymes can modulate PG hydrolytic or synthetic activity. We have previously shown that the interaction between the lytic transglycosylase RpfB and the endopeptidase RipA of mycobacteria leads to synergistic PG hydrolytic activity *in vitro* [12]. Similarly, increased PG synthesis is observed *in vitro* by the interaction of the *E. coli* PG synthase PBP1B and the structural cell division protein FtsN [29]. Despite these advances in understanding how individual interactions affect PG remodeling, a mechanism for the regulated coordination between PG hydrolytic and synthetic processes, which must occur *in vivo*, has only been theorized. In support of this theory, inactivation of PBPs with penicillin treatment in pneumococcus rapidly leads to unchecked murein hydrolase activity and bacterial lysis [30]. Furthermore, overexpression of a catalytically inactive PBP1B in *E. coli* leads to lysis of the bacterium [31], suggesting the presence of a PBP1 protein complex capable of controlling autolysin activity. Here we report the novel interaction between the *M. tuberculosis* PG hydrolase RipA and the PG synthase PBP1. This RipA-PBP1 interaction not only provides three of the four necessary PG remodeling activities, but also regulates PG remodeling by antagonizing the synergistic hydrolytic activity of the RipA-RpfB interaction. Molecularly, this is consistent with the predicted sites of action of the two hydrolases and the bonds catalyzed by PBP1 (Figure 7C).

**Figure 8:**
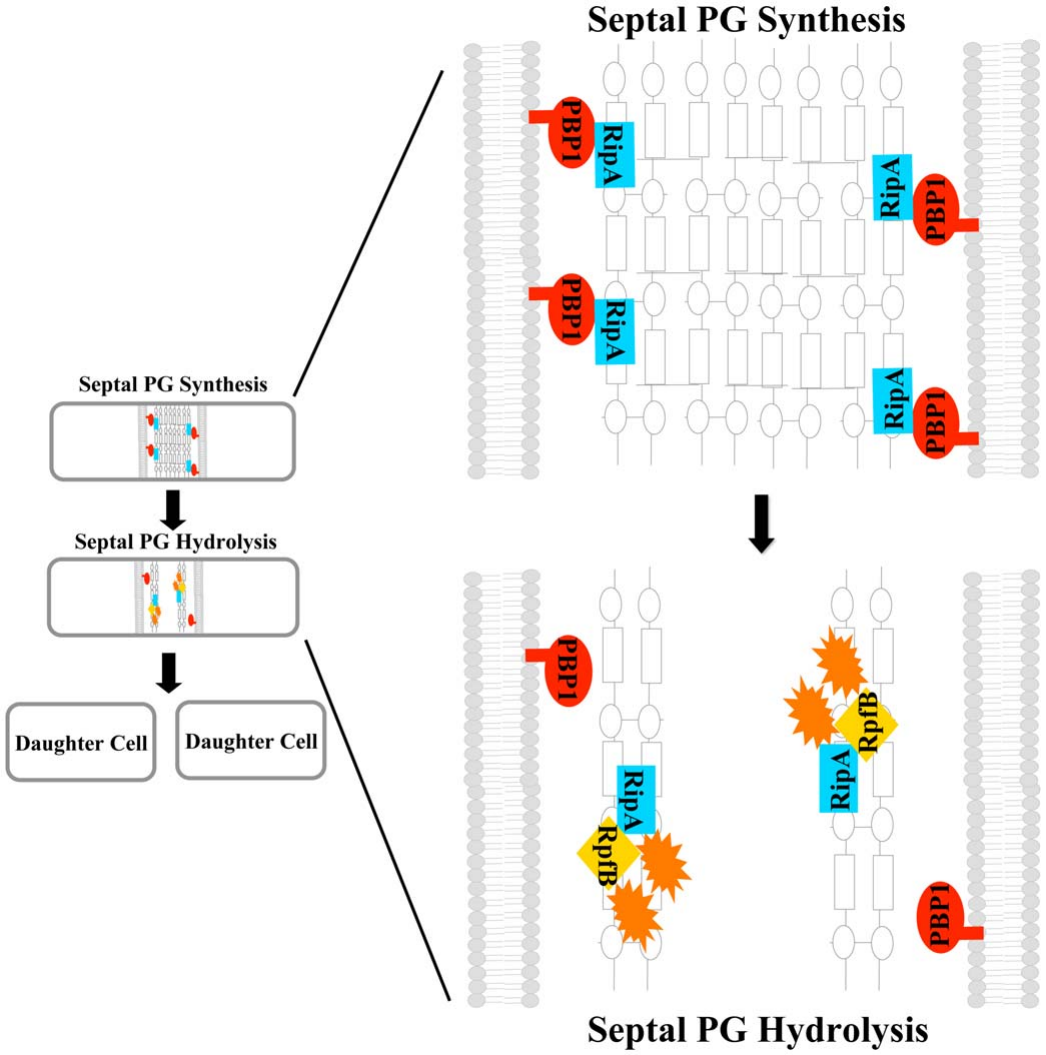
Model of RipA regulation by PBP1 during septation. A RipA-PBP1 complex that exists at the initiation of septation may synthesize a thick layer of peptidoglycan (PG) between daughter cells during the process of cytokinesis. After PG synthesis and fission are finished, RipA may exchange PBP1 for autolysin binding partners like RpfB. These new complexes are highly efficient at PG hydrolysis and will separate the cell walls of the two mature daughter cells.

There are several possibilities for how PBP1 could inhibit RipA-RpfB synergy. Given that both PBP1 and RpfB bind RipA, the most likely scenario is competition between PBP1 and RpfB for binding to RipA. Because we find that RipA interacts with PBP1 at the same C terminal 25 amino acids of RipA required for RpfB binding, PBP1 could displace RpfB. This would explain the *in vitro* antagonism between RpfB and PBP1 for activating hydrolytic activity. Furthermore, these interactions might help coordinate septal PG synthesis and division *in vivo* as shown in Figure 8. In this model, PBP1 might complex with RipA and inhibit PG hydrolysis sufficiently to allow septal PG synthesis. When septal PG is fully synthesized, RpfB may compete with PBP1 for binding to RipA, leading to the formation of a highly effective PG hydrolysis complex and the coordinated degradation of septal PG during separation of daughter cells.

PG hydrolysis experiments reported here were conducted with *M. luteus* and *Streptococcus* PG, demonstrating a general ability for the enzymes to regulate degradation of several types of PG. However, the regulation of mycobacterial PG degradation *in vivo* is likely to be more complex. Our assays for PG hydrolysis are admittedly imperfect. Events that must occur in minutes or hours in the cell require days to detect. In part, this is due to the non-physiological conditions in the assay systems. Activity might be affected by modifications to either the enzymes or the structure of peptidoglycan. In fact, the functional interactions we observe likely represent only a small portion of the regulatory interactions in the cell wall, which probably include other proteins as well as modifications of enzymes and their substrates.

Because of its localization at the septum and poles in the actinobacterium *C. glutamicum*, PBP1 is thought to be responsible for synthesis of both septal and polar PG [17], [18], [19], [23], [32]. To evaluate whether PBP1 functions similarly in mycobacteria, we sought to determine the localization of PBP1 *in vivo*. We find that PBP1-RFP localizes to the poles and septa in mycobacteria, the two primary sites of PG synthesis in mycobacteria, suggesting that PBP1 functions in both elongation and septation. It is possible that the C-terminal RFP fusion affects localization of PBP1. However, RipA also localizes to both the poles and septum of mycobacteria [11] and C-terminally tagged RipA remains functional (not shown).

PBP1 plays a critical role in PG synthesis and viability across divergent bacterial species. Depletion of the PBP1 paralogues in *C. glutamicum* results in defects in PG elongation and division [23]. Likewise, in *E. coli*, the similar PBP1A and PBP1B proteins are each dispensable for growth, but deletion of both genes is lethal, with defects in both cell elongation and septation [33]. Disruption of *ponA1* in *M. smegmatis* was previously shown to result in decreased growth and hypersensitivity to β-lactams antibiotics [34]. Clearly, in this published work, PBP1 could be disrupted and cells continued to grow. Methodological differences could account for the phenotype we see. Isolated mutants are under strong selective pressure and might easily develop compensatory mutations that permit growth. In the case of PBP1, for example, this might be due to overexpression of another PBP with partially overlapping function. In our system, cells are not under selective pressure until inducer is removed and cannot rapidly adapt to the loss of the enzyme. However, selective pressure is strong enough to rapidly select for strains that produce PBP1 even in the absence of inducer (Figure 2SA), again suggesting that loss of PBP1 is highly deleterious.

When PBP1 was provided *in trans*, the depletion strain grew like wildtype, implicating the importance of PBP1 in the depleted operon. Cells that express decreased amounts of PBP1 are small and abnormally shaped, consistent with the notion that PBP1 is involved in both elongation and septation. The bulging seen in these cells phenocopies the morphology of penicillin-treated bacteria prior to autolysin-dependent lysis [35]. This abnormal morphology is characteristic of increased and/or dysregulated PG hydrolytic activity, leading to a loss of structural integrity. This phenotype is predicted by our model, in which PBP1 is necessary for regulating PG hydrolysis, in part by restraining RipA from synergizing with RpfB (and possibly with other autolysins).

Finally, it is plausible that protein-protein interactions between different PG remodeling enzymes within cell wall complexes are universal molecular mechanisms for coordinating the different growth states of bacteria. While this work has begun to define the role of these interactions during cell division, this mechanism of action may extend beyond exponential growth. Could mycobacteria use similar regulatory systems for other growth conditions such as reactivation of dormant cells from dormancy? In *B. subtilis*, regrowth from a spore, or germination, involves several division machinery genes, including DivIVA (a MinCD regulator and chromosome partitioner protein) [36] and PrkC (a Ser/Thr protein kinase) [37]. Interestingly, mycobacterial homologues of these genes, *wag31* and *pknB*, respectively, are key regulators of division and morphology during vegetative growth [38], [39], and may serve a dual function during resuscitation of mycobacteria. Of note, the Rpf PG hydrolase family of proteins appears necessary for mycobacterial resuscitation from dormancy *in vitro* [40], [41], [42], [43], [44] and survival *in vivo* [45], [46], [47]. Given the interaction between RipA and RpfB and their synergistic function in septal PG remodeling, the Rpf proteins may represent another example of enzymes that play different biological roles during different growth states. Understanding the molecular mechanism by which vegetative PG modifying enzymes achieve cell wall homeostasis may inform us on how cells can transition between physiological states.

## Materials and Methods

### Strains and culture conditions

*E. coli* XL-1 (Stratagene) strains were used for cloning and were grown at 37°C in Luria-Bertani (LB) broth or agar and supplemented with kanamycin (50 µg/ml), ampicillin (100 µg/ml), hygromycin (100 µg/ml) or zeocin (25 µg/mL) when appropriate. *E. coli* BL21 (DE3) (Stratagene) was used for production of recombinant proteins from the pET41a (Novagen) or pMal (New England Biolabs) vectors for GST or MBP fusions, respectively. *Mycobacterium smegmatis* (mc^2^155) was grown at 37°C in Middlebrook 7H9 broth supplemented with ADC (albumin-dextrose-catalase) and 0.05% Tween80 and kanamycin (25 µg/ml) or hygromycin (50 µg/ml) when appropriate. *Saccharomyces cerevisiae* strains PJ69–4A (*MATA trp1–901 leu2–3,112 ura3–52his3–200 gal4 gal80 LYS2*::*GAL1-HIS3 GAL2-ADE2 met2*::*GAL7-lacZ*) was grown at 30°C in appropriate selective media and transformed according to the Clontech Matchmaker manual or using the Zymo EZ Kit (Zymo Research).

### Yeast two-hybrid screen

We fused DNA encoding the C-terminal 123 amino acids of the *M. tuberculosis* allele of RipA to DNA encoding the yeast GAL4 DNA binding domain (BD-RipA) in the pAS4 vector (similar to pAS2, but with a uracil marker rather than tryptophan) and screened against a random library of *M. tuberculosis* gene fragments fused to DNA encoding the GAL4 activation domain (AD) using the Matchmaker System (Clontech) as previously published [48]. Interactions were required to grow on plates lacking histidine or adenine and produce β-galactosidase. Potential candidates were tested for nonspecific interaction with the human Lamin protein. Further mapping of interacting regions was conducted similarly, but with known gene fragments. Growth was determined by visualizing the density of growth on selective plates and was categorized as ‘+++’ (strong), ‘++’ (moderate), ‘+’ (minimal, but evident), and ‘–’ (lacking).

### β-galactosidase liquid assay

Three independent cultures of each yeast strain were assayed for β-galactosidase activity using ONPG (o-Nitrophenyl-beta-D-Galactopyranoside) as substrate, according to the Clontech Matchmaker manual.

### Recombinant protein production

DNA encoding the C-terminal 283 amino acids of RipA was cloned into the pMal-C2X MBP expression vector (New England Biolabs) as well as the pET41a GST expression vector, while DNA encoding the 259 amino acids of the C-terminal of PBP1 or DNA encoding the 70 amino acid conserved region of RpfB was cloned into the pET41a GST expression vector (Novagen). The *E. coli* expression strain, BL21(DE3) was used to synthesize each protein following the Novagen manual protocol. Protein concentrations were measured using the Bradford assay, normalized, and confirmed by Coomassie Blue-stained polyacrylamide gels.

### Co-precipitation assay

DNA encoding the C terminal 259 amino acid portion of *M. tuberculosis* PBP1 was cloned into pET41a to create a GST fusion. DNA encoding the C terminal 283 amino acids of M. tuberculosis RipA were cloned into the pMalC2x vector to create a MBP fusion. Bl21 *E. coli* were co-transformed with both PBP1 and RipA fusion plasmids. As a control, the RipA-MBP plasmid was also co-transformed with an empty pET41a GST plasmid. Cells were grown to an OD of 0.5, induced with 1mM IPTG at 30°C for 3 hours and lysed by sonication for 10 seconds, 15 cycles in HEPES lysis buffer (25 mM HEPES, 50 mM KCl, 5mM MgCl_2_, pH 7.5). Recombinant GST fusion proteins were precipitated with Glutathione Sepharose 4B resin (Amersham Biosciences) for 1 hour at 4°C, rotating. The resin was then washed 3 times with cold 1× PBS +1% Triton X-100. Recombinant +and co-precipitating proteins were eluted with glutathione elution buffer (10mM reduced glutathione, 50mM Tris-HCl, pH 8.0) at 25°C, 15 minutes. Also, equimolar amounts of purified and normalized GST or GST-PBP1 proteins were combined with equimolar amounts of normalized MBP or MBP-RipA proteins in 1.5 ml tubes containing 500 µl PBS. The protein mixture was gently rocked at 4°C for 4 to 15 hours. Before further purification, 60 µl of mixture was removed and saved as a loading control. From the remaining mixture, MBP proteins were purified using amylose resin or GST proteins were purified using sepharose (New England Biolabs) as per directions. Co-purifying proteins and loading controls were detected using immunoblotting with a GST or MBP polyclonal antibody at 1∶10,000 dilution.

### Immunoblotting

Protein samples were combined with 4× Laemmli's SDS PAGE buffer and boiled at 100°C for 5 minutes. Proteins were separated on 8% Tris-tricine polyacrylamide gels by electrophoresis, transferred to nitrocellulose, and probed with anti-sera against MBP (New England Biolabs) or GST using standard techniques.

### Preparation and FITC-labeling of cell wall material

*Streptomyces* peptidoglycan and lyophilized *M. luteus* cell wall were both obtained from Sigma. The fluorescein isothiocyanate (FITC)-labeled bacterial cell wall was prepared by covalently linking FITC to amine groups in the cell wall. 10 mg FITC (Molecular Probes) was used to label 10 mg of insoluble peptidoglycan or cell wall material following the protocol from Protocols in Protein Science (adapted from Molecular Probe notes).

### Enzyme assay

Recombinant *M. tuberculosis* proteins were incubated with several FITC-labeled cell wall substrates and assayed for activity by measuring FITC release. 25µg of Rpf, PBP1 or RipA alone or in various combinations, was added to 25 µl of 2 mg/ml substrate and 25 µl 4× reaction buffer (50 mM Tris, 10mM MgCl, 50 mM KCl, 2mM MnCl, 0.01% Chaps, 100 mM KH_2_PO_4_, pH 5.75). The final volume was brought to 100 µl with H_2_O. Similar combinations with GST were also tested. GST alone, as well as buffer alone, was used to determine background release of FITC. After incubating at 30°C with enzyme and buffer for 3–5 days, the insoluble substrate was centrifuged (5 minutes at 18,000×g) and soluble FITC remaining in the supernatant was measured with filters for excitation 485 nm and emission 538 nm. Significance was determined using one-tailed, unpaired t-tests using Prism software.

### Generation of depletion strains

The depletion strain was generated as previously described [12], [49]. Briefly, *M. smegmatis*, with the tetracycline repressor gene integrated into the *attB* site, was transformed in the presence of anhydrotetracylcine with a suicide vector containing the first 600 nucleotides of *M. smegmatis ponA1* gene under control of the tetracycline operator/promoter system (Ptet). Transformants were selected for hygromycin resistance. Appropriate recombination was confirmed using forward primers to Ptet and PponA1 (native *ponA1* promoter) paired with a reverse primer to the 3′ end of *ponA1*. The presence of a product of appropriate size for the former and lacking in the latter, confirmed the desired strain.

### Depletion strain growth

The *ponA1* (PBP1) depletion strain was initially grown in 7H9 media containing kanamycin (selecting for TetR) and hygromycin (selecting for inserted pTet) as well as anhydrotetracycline (ATC). Once cultures reached late log-phase or stationary phase, they were centrifuged (2500×g for 5 minutes), washed once with PBS, and resuspended in media with varying amounts of ATC.

### Complementation analysis

*M. smegmatis ponA1* was synthesized by Genscript (Piscataway, NJ) and cloned under the control of the *M. tuberculosis* GroEL2 promoter. Monomeric RFP was cloned into the complementation vector alone or downstream of *ponA1*, as a control to confirm expression from this promoter. Complementation vectors expressing RFP alone or PBP1 with RFP were electroporated into the *ponA1* depletion strain and transformants selected on hygromycin, kanamycin and zeocin supplemented with 100ng/mL ATC. Complemented strains were grown in 7H9 with 100 ng/mL ATC until log phase, and then diluted to an OD of approximately 0.0002 and grown in media containing various concentrations of ATC.

### Real time PCR

*M. smegmatis* strains were grown with either 50 ng/mL or no anhydrotetracycline and all samples were collected at mid log phase. *ponA1* expression was measured using the following primers:

5′ GGAGGCATCAAGGCGTACTA; 5′ AACACCTTGAACGACGAACC.

*ponA1* levels were normalized to *sigA* expression, which was measured using the following primers:

5′ AAGACACCGACCTGGAACTC; 5′ AGCTTCTTCTTCCTCGTCCTC.

Samples were prepared according to the mechanical disruption protocol in the RNA Protect Bacteria Reagent handbook (Qiagen, Valencia, CA) and cell pellets stored at −80C. Disruption was achieved with three, 1 minute beadbeating cycles. RNA was isolated using the RNeasy Mini Kit (Qiagen), but with an additional DNAse treatment on the column before elution and a second DNAse digestion with Turbo DNase according to manufacturer's instructions (Ambion, Foster City, CA). Reverse transcription of the RNA was carried out using the High Capacity cDNA Reverse Transcription Kit (Applied Biosystems, Foster City, CA). Quantitative PCR utilized Power SyBr green PCR master mix (Applied Biosystems) and reactions were run and analyzed on a Step One Plus real time system (Applied Biosystems).

### Microscopy and imaging

*M. smegmatis* strains were centrifuged at 2500×g for 2 minutes, washed with 1ml PBS, and resuspended in 20 µl of PBS containing 50µM TMA-DPH for staining membranes. Samples were imaged using a Nikon TE-200E microscope with a 100× (NA 1.4) objective and captured with an Orca-II ER cooled CCD camera (Hamamatsu, Japan). Shutter and image acquisition were controlled using Metamorph Software (Molecular Devices). Final images were prepared using Adobe Photoshop 7.0.

## Supporting Information

Figure S1 Diagram of *ponA1* and *ponA2* operons. *ponA1* (*rv0050* of *M. tuberculosis* and MSMEG6900 of *M. smegmatis*), is the first gene in an operon with two other genes of unknown function. *rv0051* (MSMEG6899) encodes a conserved transmembrane protein with 24% identity to GPI mannosyl-transferase with a DXD motif common in glycosyltransferases that utilize nucleotide sugars and *rv0052* (MSMEG6898) encodes a conserved hypothetical protein. There are two paralogues of *ponA* in both *M. tuberculosis* and *M. smegmatis*, *ponA1* and *ponA2*, similar to other bacteria. *ponA2* (*rv3682*) of *M. tuberculosis* encodes the first gene in a predicted operon with two other genes. *rv3863* encodes a conserved secreted phosphohydrolase, possibly involved in histidine biosynthesis. *rv3684* encodes a protein with homology to cysteine synthases. Arrows in black represent encoding genes of the *ponA* predicted operon, while grey arrows represent the next encoding gene on either side of the operon.
(0.49 MB TIF)

Figure S2 PBP1 depletion strain escape from regulation (A) Growth of the PBP1 depletion strain was analyzed by optical density in the presence and absence of the inducer, anhydrotetracycline (ATC). (B) *ponA1* expression was analyzed by RT-PCR in PBP1 depletion strains grown in the presence of inducer (50 ng/ml ATC) and in three independent cultures grown without inducer (no ATC). The no ATC cultures began to grow at late time points (∼50 hours). All four strains were taken at mid exponential phase for PBP1 transcript analysis and normalized by *sigA* levels.
(0.27 MB TIF)
