# Supplementary figures and images for: Interaction and Modulation of Two Antagonistic Cell Wall Enzymes of Mycobacteria
Source: PLoS Pathog. 2010 Jul 29;6(7):e1001020. doi: 10.1371/journal.ppat.1001020 (PMC2912383; doi:10.1371/journal.ppat.1001020)

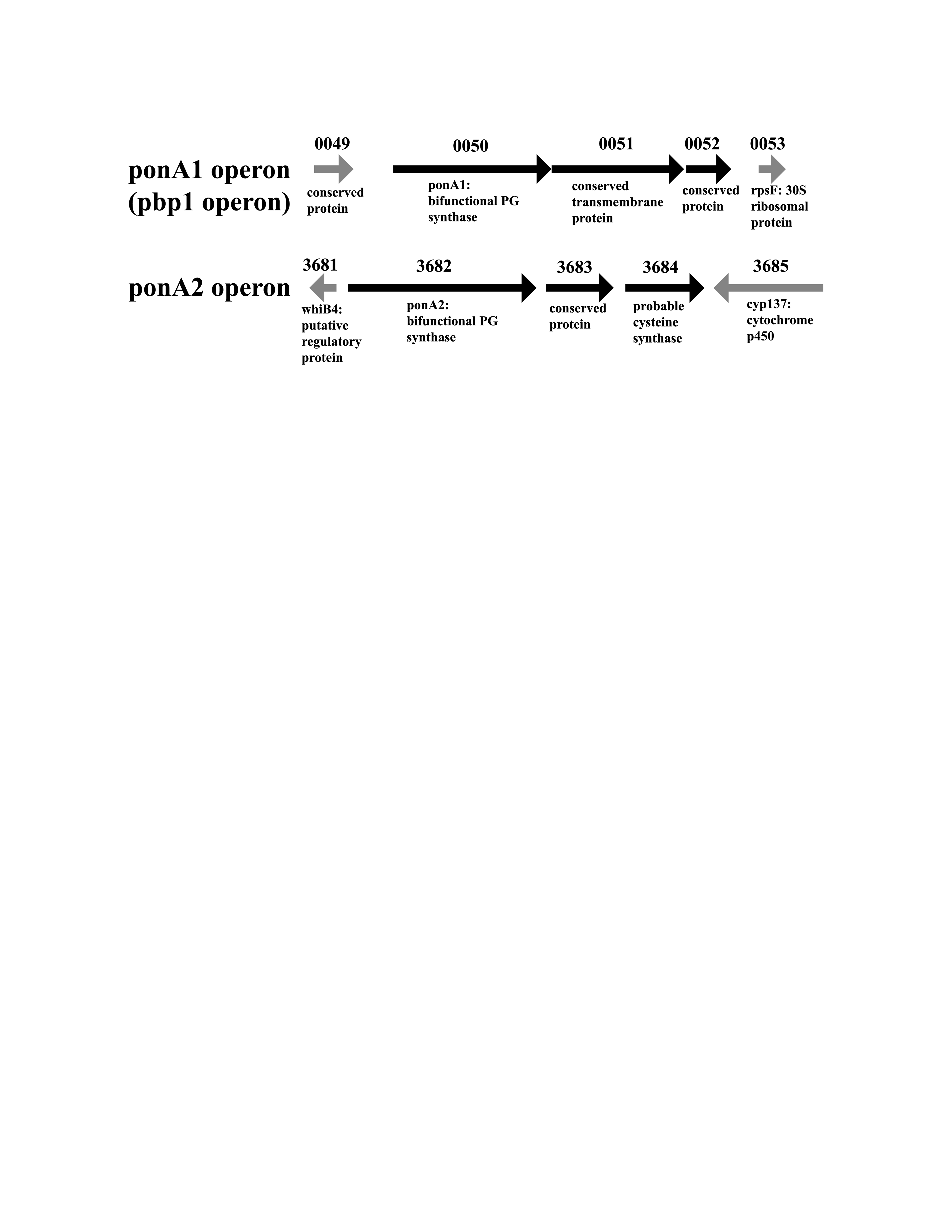

Supplement: Figure S1 — Diagram of ponA1 and ponA2 operons. ponA1 (rv0050 of M. tuberculosis and MSMEG6900 of M. smegmatis), is the first gene in an operon with two other genes of unknown function. rv0051 (MSMEG6899) encodes a conserved transmembrane protein with 24% identity to GPI mannosyl-transferase with a DXD motif common in glycosyltransferases that utilize nucleotide sugars and rv0052 (MSMEG6898) encodes a conserved hypothetical protein. There are two paralogues of ponA in both M. tuberculosis and M. smegmatis, ponA1 and ponA2, similar to other bacteria. ponA2 (rv3682) of M. tuberculosis encodes the first gene in a predicted operon with two other genes. rv3863 encodes a conserved secreted phosphohydrolase, possibly involved in histidine biosynthesis. rv3684 encodes a protein with homology to cysteine synthases. Arrows in black represent encoding genes of the ponA predicted operon, while grey arrows represent the next encoding gene on either side of the operon. (0.49 MB TIF) [file ppat.1001020.s001.tif]

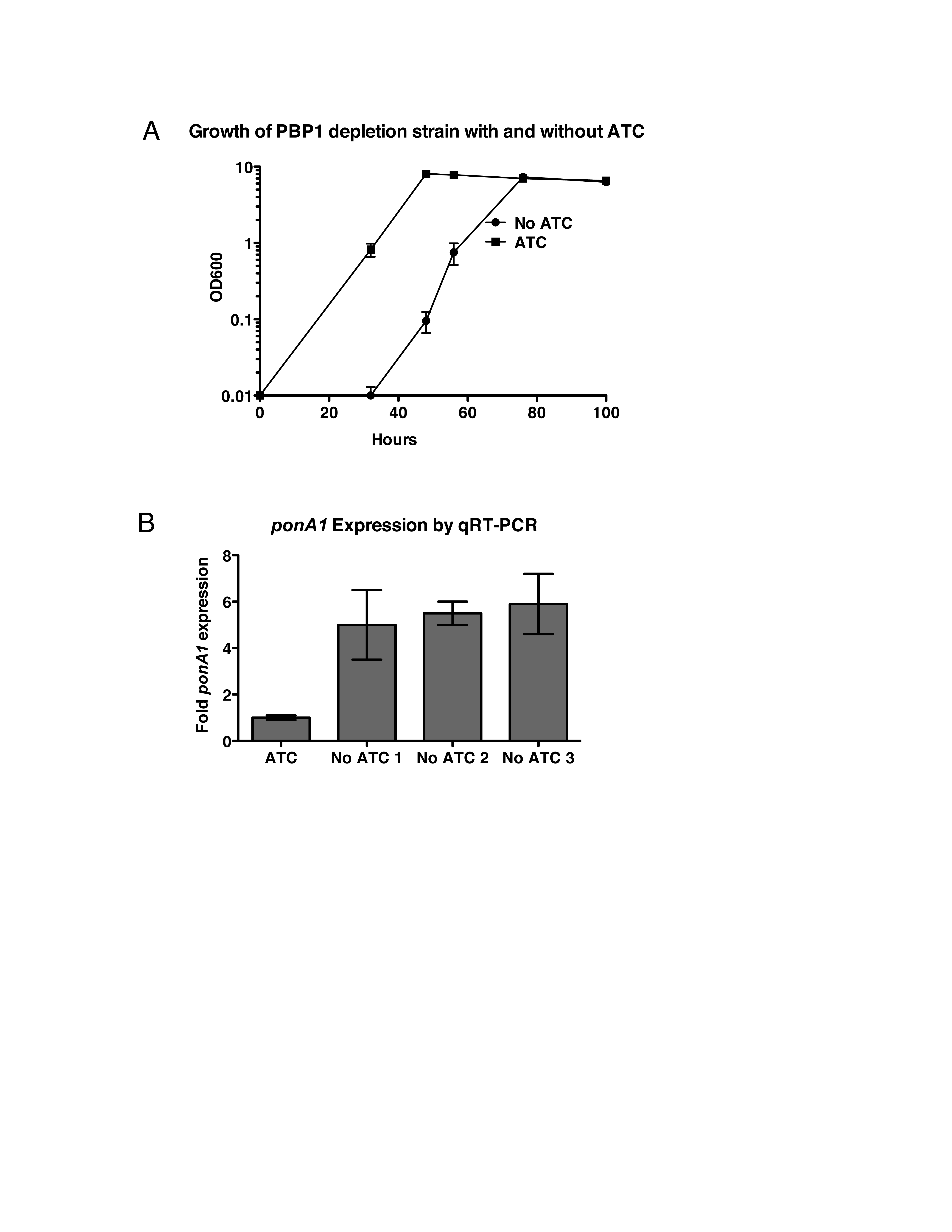

Supplement: Figure S2 — PBP1 depletion strain escape from regulation (A) Growth of the PBP1 depletion strain was analyzed by optical density in the presence and absence of the inducer, anhydrotetracycline (ATC). (B) ponA1 expression was analyzed by RT-PCR in PBP1 depletion strains grown in the presence of inducer (50 ng/ml ATC) and in three independent cultures grown without inducer (no ATC). The no ATC cultures began to grow at late time points (∼50 hours). All four strains were taken at mid exponential phase for PBP1 transcript analysis and normalized by sigA levels. (0.27 MB TIF) [file ppat.1001020.s002.tif]
